# Supplementary material for: Heat adaptation of phage T7 under an extended genetic code
Source: Virus Evol. 2021 Dec 1;7(2):veab100. doi: 10.1093/ve/veab100 (PMC8923235; doi:10.1093/ve/veab100)
Supplement: veab100_Supp [file veab100_supp.zip › Sequencing_Analysis.zip!/Sequencing_Analysis/A3/evidence/SNP_7.html]

BRESEQ :: Evidence


| Predicted mutation | | | | | | |
| --- | --- | --- | --- | --- | --- | --- |
| evidence | seq id | position | mutation | annotation | gene | description |
| RA | NC\_001604.1 | 24,238 | A→G | intergenic (–/–) | – / – | –/– |

| Read alignment evidence... | | | | | | | | | | | |
| --- | --- | --- | --- | --- | --- | --- | --- | --- | --- | --- | --- |
|  | seq id | position | | ref | new | freq | score (cons/poly) | reads | annotation | genes | product |
| \* | NC\_001604.1 | 24,238 | 0 | A | G | 98.5% | 4591.6 / NA | 1549 | intergenic (–/–) | *–/–* | –/– |
| Reads supporting (aligned to +/- strand):  **ref** base A (2/1);  **major** base G (834/687);  **minor** base T (19/4);  **total** (857/692) | | | | | | | | | | | |
| Fisher's exact test for biased strand distribution *p*-value = 9.76e-03 | | | | | | | | | | | |
| Kolmogorov-Smirnov test that lower quality scores support variant *p*-value = 1.00e+00 | | | | | | | | | | | |

Only 100 of 1549 total aligned reads displayed.  

`ACGCAATGGGCCACGGTGGTCTTCGCCCAGAAGCTGCTGGTGCAGTGGTTTTCAAAGTGGAGTAATGCTGGGGGTGGCCTCAACGGTCGCTGCTAGTCCCGAAGAGGCGAGTGTTACTTCAACAGAAGAAACCTTAACGCCAGCACAGGAGGCCGCACGCACCCGCGCTGCTAACAAAGCCCGAAAGGAAGCTGAGTTGGCTGCTGCCACCGCTGAGCAATAACTAGCATAACCCCTTGGGGCCTCTAAACGGGTCTTGAGGGGTTTTTTGCTGAAAGGAGGAACTATATGCGCTCATACGATATGAACGTTGAGACTGCCGCTGAGTTATCAGCTGTGAACGACATTCTGGCGTCTATCGGTGAACCTCCGGTATCAACGCTGGAAGGTGACGCTAACGCAGATGCAGCGAACGCTCGGCGTATTCT‑CAACAAGATTAACCGACAGATTCAATCTCGTGGATGGACGTTCAACATTGAGGAAGGCATAACGCTACTACCTGATGTTTACTCCAACCTGATTGTATACAGTGACGACTATTTATCCCTAATGTCTACTTCCGGTCAATCCATCTACGTTAACCGAGGTGGCTATGT  >  NC_001604.1/23940‑24535`  

                                                                                                                                                                                                                                                                                                          `|`                                                                                                                                                                                                                                                                                                            
`cagCAATGGGCCACGGTGGTCTTCGCCCAGAAGCTGCTGGTGCAGTGGTTTTCAAAGTGGAGTAATGCTGGGGGTGGCCTCAACGGTCGCTGCTAGTCCCGAAGAGGCGAGTGTTACTTCAACAGAAGAAACCTTAACGCCAGCACAGGCGGCCGCACGCACCCGCGCTGCTAACAAAGCCCGAAAGGAAGCTGAGTTGGCTGCTGCCACCGCTGCGCAATCACTAGCATAACCCCTTGGGGCCTCTAAACGGGTCTTGAGGGGTTTTTTGCTGACAGGAGGAACTATATGCGCTCATGCg                                                                                                                                                                                                                                                                                                          >  1:19981/3‑301 (MQ=255)`  
       `gggCCACGGTGGTCTTCGCCCAGAAGCTGCTGGTGCAGTGGTTTTCAAAGTGGAGTAATGCTGGGGGTGGCCTCAACGGTCGCTGCTAGTCTCGAAGAGGCGAGTGTTACTTCAACAGAAGAAACCTTAACGCCAGCACAGGAGGCCGCACGCACCCGCGCTGCTAACAAAGCCCGAAAGGAAGCTGAGTTGGCTGCTGCCACCGCTGAGCAATCACTAGCATAACCCATTGGGGCCTCTAAACGGGTCTTGAGGGGTTCTTTGCTGAAAGGAGGCACTATATGCGCTCATGCGATATGaa                                                                                                                                                                                                                                                                                                   >  1:256570/1‑301 (MQ=255)`  
       `gggCCACGGTGGTCTTCGCCCAGAAGCTGCTGGTGCAGTGGTTTTCAAAGTGGAGTAATGCTGGGGGTGGCCTCAACGGTCGCTGCTAGTCCCGAAGAGGCGAGTGTTACTTCAACAGAAGAAACCTTAACGCCAGCACAGAAGGCCGCACGCACCCGCGCTGCTAACAAAGCCCGAAAGGAAGCTGAGTTGGCTGCTGCCACCGCTGAGCAATCACTAGCATAACCCCTTGTGGCCTCTAAACGGGTCTTGAGGGGTTTTTTGCTGAAAGGAGGAACTATATGCGCTCATGCGCTATGaa                                                                                                                                                                                                                                                                                                   >  1:249607/1‑301 (MQ=255)`  
                `tggtctgcgtgacgAAGCTGCTGGTGAAGTGGTTTGCAAAGGGGAGTCAGGCTGGGGGGGGCCTCAACGGTCGCTGCTAGTACCGAAGAGGCGAGTGTTACTTAAACAGAAGAAACCTTAACGCCAGCACAGGAGGCCGACCGCACCAGAGAGGCTAACCAAGCCCGAAAGGAAGCTGAGTTGGCTGTTTACACCGCTGAGCAATCACTAGCAGAACCCCTTGGTGCTTCTAAACGGGTCGTGAGGGGTTTTTTGCTGAAAGGAGGAACGATATGCGCTCATGCGAGATGAACGTTgagat                                                                                                                                                                                                                                                                                          <  1:83617/288‑2 (MQ=255)`  
                           `cAGAAGCTGCTGGTGCAGTGGTTTTCAAAGTGGAGTAATGCTGGGGGGGGCCTCAACGGTCGCTGCTAGTCCCGAAGAGGCGAGTGTTACTTCAACAGAAGAAACCTTAACGCCAGCACAGGAGGCCGCACGCACCCGCGCTGCTAACAAAGCCCGAAAGGAAGCTGAGTTGGCTGCTGCCACCGCTGAGCAATCACTAGCATAACCCCTTGGGGCCTCTAAACGGGTCTTGAGGGGTTTTTTGCTGAAAGGAGGAACTATATGCGCTCATGCGATATGAACGTTGAGACTGCCGCTGAGt                                                                                                                                                                                                                                                                               <  1:274186/301‑1 (MQ=255)`  
                             `gAAGCTGCTGGTGCAGTGGTTTTCAAAGTGGAGTAATGCTGGGGGTGGCCTCAACGGTCGCTGCTAGTCCCGAAGAGGCGAGTGTTACTTCAACAGAATAAACCTTAACGCCAGCACAGGAGGCCGCACGCACCTGCGCTGCTAACAAAGCCCGAAAGGAAGCTGAGTTGGCTGCTGCCACCGCTGAGCAATCACTAGCATAACCCATTGGGGCCTCTAAACGGGTCTTGAGGGGTTTTTTGCTGAAAGGAGGAACTATATGCGCTCATGCGATATGAACGTTGAGACCGCCCCTGAGTTa                                                                                                                                                                                                                                                                             >  1:171306/1‑301 (MQ=255)`  
                                  `gctcggagatgtgtataagagacaggAGTAATGCTGGGGGTGGCCTCAACGGTCGCTGCTAGTCCCGAAGAGGCGAGTGTTACTTCAACAGAAGAAACCTTAACGCCAGCACAGGAGGCCGCACGCACCCGCGCTGCTAACAAAGCCCGAAAGGAAGCTGAGTTGGCTGCTGCCACCGCTGAGCAATCACTAGCATAACCCCTTGGGGCCTCTAAACGGGTCTTGAGGGGTTTTTTGCTGAAAGGAGGAACTATATGCGCTCATGCGATATGAACGTTGAGACTGCCGCTGAGTTATCAGc                                                                                                                                                                                                                                                                        <  1:168158/277‑1 (MQ=255)`  
                                   `gctgGTGCAGTGGTTTTCAAAGTTGAGTAATGCTGGGGGTGGCCTCAACGGTCGCTGCTAGTCCCGAAGAGGCGAGTGTTACTTCAACAGAAGAAACCTTAACGCCAGCACAGGAGGCCGCACGCACCCGCGCTGCTAACAAAGCCCGAAAGGAAGCTGAGTTGGCTGCTGCCACCGCTGAGCAATCACTAGCATAACCCCTTGGGGCCTCTAAACGGGGCTTGAGTGGTTTTTTGCTGAAGGGAGGAACTATCTGCGCTCATGCGATCTGAACTTTGAGACTGCCGCTGAGTTATCAGCt                                                                                                                                                                                                                                                                       >  1:162982/1‑301 (MQ=255)`  
                                    `ctgGTGCAGTGGTTTTCAAAGTGGAGTAATGCGGGGGGTGGCCTGAACGGTCGCTGCTAGTCCCGAAGAGGCGAGTGTTACTTCAACAGAAGAAACCTTAACGCCAGCACAGGAGGCCGCACGCACCCGCGCTGCTAACAAAGCCCGAAAGGAAGCTGAGTTGGCTGCTGCCACCGCTGAGCAATCACTAGCATAACCCCTTGGGGCCTCTAAACGGGTCTTGAGGGGTTTTTTGCTGAAAGGAGGAACTATATGCGCTCATGCGATATGAACGTTGAGACTGCCGCTGAGTTATCAGCtg                                                                                                                                                                                                                                                                      <  1:86781/301‑1 (MQ=255)`  
                                      `ggTGCAGTGGTTTTCAAAGTGGAGTAATGCTGGGGGTGGCCTCAACGGTCGCTGCTAGTCCAGAAGAGGCGAGTGTTACTTCAACAGAAGAAACCTTAACGCCAGCACAGGAGGCCGCACGCACCCGCGCTGCTAACAAAGCCCGAAAGGAAGCTGAGTTGGCTGCTGCCACCGCTGAGCAATCACTAGCATAACCCCTTGGGGCCTCTAAACGGGTCTTGAGGGGTTTTTTGCTGAAAGGAGGAACTATATGCGCTCAAGCGATATGCACGTTGAAAATGACGCTGAGTTATCAGCtgtt                                                                                                                                                                                                                                                                    >  1:191157/1‑300 (MQ=255)`  
                                         `gCAGTGGTTTTCAAAGTGGAGTAATGCTGGGTGTGGCCTAAACTGTCGCTGCTAGTCCCGAAGAGGCGAGAGTTAATTCAACAGAAGAAACCTTAACGCCAGCACAGGAGGACGCAAGCACCCGCGCTGGTAACAAAGCCCGAAAGGAAGCTGAGTTGGATGCTGCCACCGCAGAGCAATCAATAGCATAACCCCTTGGGGGCTCTAAAAGGGTCGTGAGGGGATTTTTGCTGAAAGGAGGAACTATATGCGCTCATGCGATATGAACGTTGAGACTGCCGCTGAGTTATCAGCTGTGAAc                                                                                                                                                                                                                                                                 <  1:253736/301‑1 (MQ=255)`  
                                         `gCAGGGGTTGTCAAAGTGGAGTAATGCTGGGGGTGGCCTCAACGGTCGCTGCTAGTCCCGAAGAGGCGAGTGTTACTTCAACAGAAGAAACCTTAACGCCAGCACAGGAGGCCGCACGCACCCGCGCTGCTAACAAAGCCCGAAAGGAAGCTGAGTTGGCTGCTGCCACCGCTGAGCAATCACTAGCATAACCCCTTGGGGCCTCTAAACGGGTCTTGAGGGGTTTTTTGCTGAAAGGAGGAACTATATGCGCTCATGCGATATGAACGTTGAGACTGCCGCTGAGTTATCAGCTGTGAAc                                                                                                                                                                                                                                                                 <  1:52890/301‑1 (MQ=255)`  
                                                `ttttCAAAGTGGAGTAATGCTGGGGGTGGCCTCAACGGTCGCTGCTAGTCCCGAAGAGGCGAGTGTTACTTCAACAGAAGAAACCTTAACGCCAGCACAGGAGGCCGCACGCACCCGCGCTGCTAACAAAGCCCGAAAGGAAGCTGAGTTGGCTGCTGCCACCGCTGAGCAATCACTAGCATAACCCCTTGGGGCCTCTAAACGGGTCTTGAGGGGTTTTTTGCTGAAAGGAGGAACTATATGCGCTCATGCGATATGAACGTTGAGACTGCCGCTGAGTTATCAGCTGTGAACGACATTc                                                                                                                                                                                                                                                          <  1:244107/301‑1 (MQ=255)`  
                                                     `aaggTGGAGTAATGCTGGGGGTGGCCTCAACGGTCGCTGCTAGTCCCGAAGAGGCGAGTGTTACTTCAACAGAAGAAACCTTAACGCCAGCACAGGAGGCCGCACGCACCCGCGCTGCTAACAAAGCCCGAAAGGAAGCTGAGTTGGCTGCTGCCACCGCTGAGCAATCACTAGCATAACCCCTTGGGGCCTCTAAACGGGTCTTGAGGGGTTTTTTGCTGAAAGGAGGAACTATATGCGCTCATGCGATATGAACGTTGAGACTGCCGCTGAGTTATCAGCTGTGAACGACATTCTGGCg                                                                                                                                                                                                                                                     <  1:142562/298‑1 (MQ=255)`  
                                                        `gTGGAGTAATGCTGGGGGTGGCCTCAACGGTCGCTGCTAGTCCCGAAGAGGCGAGTGTTACTTCAACAGAAGAAACCTTAACGCCAGCACAGGAGGCCGCACGCACCCGCGCTGCTAACAAAGCCCGAAAGGAAGCTGAGTTGGCTGCTGCCACCGCTGAGCAATCACTAGCATAACCCCTTGGGGCCTCTAAACGGGTCTTGAGGGGTTTTTTGCCGAAAGGAGGAACTATATGCGCTCATGCGATATGAAAGTTGAGACTGCCGCTGAGTTATCAGCTGTGAACGACATTCTGGCGTCt                                                                                                                                                                                                                                                  >  1:318791/1‑301 (MQ=255)`  
                                                          `ggAGTAATGCTGGGGGTGGCCTCAACGGTCGCTGCTAGTCCCGAAGAGGCTAGTGTTACTTCAACAGAAGAAACCTTAACGCCAGCACAGGAGGCCGCACGCACCCGCGCTGCTAACAAAGCCCGAAAGGAAGCTGAGTTGGCTGCTGCCACCGCTGAGCAATTACTAGCATAACCCCTTGGGGCCTCTAAACGGGTCTTGAGGGGTTTTTTGCTGAAATGAGGAACTATATGCGCTCATGCGATATGAACGTTGAGACTGCCGCTGAGTTATCAGCTGTGAAcctgtctctcatacacat                                                                                                                                                                                                                                                >  1:71015/1‑284 (MQ=255)`  
                                                           `gAGTAATGCTGGGGGTGGCCTCAACGGTCGCTGCTAGTCCCGAAGAGGCGAGTGTTACTTCAACAGAAGAAACCTTAACGCCAGCACAGGAGGCCGCACGCACCCGCGCTGCTAACAAAGCCCGAAAGGAAGCTGAGTTGGCTGCTGCCACCGCTGAGCAATCACTAGCATAACCCCTTGGGGCCTCTAAACGGGTCTTGAGGGGTTTTTTGCTGAAAGGAGGAACTATATGCGCTCATGCGATATGAACGTTGAGACTGCCGCTGAGTTATCAGCTGTGAACGACATTCTGGCGTCTATc                                                                                                                                                                                                                                               >  1:260427/1‑301 (MQ=255)`  
                                                               `aaTGCTGGGGGTGGCCTCAACGGTCGCTGCTAGTCCCGAAGAGGCGAGTGTTACTTCAACAGAAGAAACCTTAACGCCAGCACAGGAGGCCGCACGCACCCGCGCTGCTAACAAAGCCCGAAAGGAAGCTGAGTTGGCTGCTGCCACCGCTGAGCAATCACTAGCATAACCCCTTTGGGCCTCTAAACGGGTCTTGAGGGGTTTTTTGCTGAAAGGAGGAACTATATGCGCTCATGCGATATGAACGTTGAGACTGCCGCTGAGTTATCAGCTGTTAACGACATTCTGGCGTCTATCGGTg                                                                                                                                                                                                                                           >  1:232995/1‑301 (MQ=255)`  
                                                                `aTGCTGGTGGTGGCCTCAACGGTCGCTGCTAGTCCCGAAGAGGCGAGTGTTACTTCAACAGAAGAAACCTTAACGCCAGCACAGGAGGCCGCACGCACCCGCGCTGCTAACAAAGCCCGAAAGGAAGCTGAGTTGGCTGCTGCCACCGCTGAGCAATCACTAGCATAACCCCTTGGGGCCTCTAAACTGGTCTTGAGGGGTTTTTTGCTGAAAGGAGGAACTATATGCGCTCATGCGATATGAACGTTGAGACTGCCGCTTAGTTATCAGCTGTGAACGACATTCTGGCGTCTATCGGTGa                                                                                                                                                                                                                                          >  1:90669/1‑301 (MQ=255)`  
                                                                  `gCTGTGGGTGGCCTCAACGGTCGCTGCTAGTCCCGAAGAGGCGAGTGTTACTTCAACAGAAGAAACCTTAACGCCAGCACAGGAGGCCGCACGCACCCGCGCTGCTAACAAAGCCCGAAAGGAAGCTGAGTTGGCTGCTGCCACCTCTGAGCAATCACTAGCATAACCCCTTGGGGCCTCTAAACGGGTCTTGAGGGGTTTTTTGCTGAAAGGAGGAACTATATGCGCTCATGCGATATGAACGTTGAGACTGCCGCTGCGTTATCAGCTGTGAACGACATTCTGGCGTCTATCTGTCAAc                                                                                                                                                                                                                                        >  1:110947/1‑301 (MQ=255)`  
                                                                    `ggggggTGGCCTCAACGGTCGCTGCTAGTCCCGAAGAGGCGAGTGTTACTTCAACAGAAGAAACCTTAACGCCAGCACAGGAGGCCGCACGCACCCGCGCTGCTAACAAAGCCCGAAAGGAAGCTGAGTTGGCTGCTGCCACCGCTGAGAAATCACTAGCATAACCCCTTGGGGCCTCTAAACGGGTCTTGAGGGGTTTTTTGCTGAAAGGAGGAACTATATGCGCTCATGCGATATGAACGTTGAGACTGCCGCTGAGTTATCAGCTGTGAACGACATTCTGGCGTCTATCGGTGAACCt                                                                                                                                                                                                                                      <  1:88527/300‑1 (MQ=255)`  
                                                                      `ggggTGGCCTCAACGGTCGCTGCTAGTCCCTAAGAGGCGAGTGTTACTTCAATAGAAGAAACCTTAACGCCAGCACAGGAGGCCGCACGCACCCGCGCTGCTAACAAAGCCCGAAAGGAAGCTGAGTTGGCTGCTGCCACCGCTGAGCAATCACTAGCATAACCCCTTGGGGCCTCTAAACGGGTCTTGAGGGGTTTTTTGCTGAAAGTAGGAACTATATGCGCTCATGCGATATGAACGTTGAGACTGCCGCTGAGTTATCAGCTGTGAACGACATTCTGGctgtctcttctacacatct                                                                                                                                                                                                                                    >  1:193390/1‑283 (MQ=255)`  
                                                                          `gggCCTCAACGGTAGCTGCTAGTCCCGAAGAGGCTAGTGTTACTTCAACAGAAGAAACCTTAACGCCAGCACAGGAGGCCGCACGCACCCGCGCTGCTAACAAAGCCCGAAAGGAAGCTGAGTTGGCTGCTGCCACCGCTGAGCAATCACTAGCATAACCCCTTGGGGCCTCTAAACGGGTCTTGAGGGGTTTTTTGCTGAAATGAGGAACTATATGCGCTCATGCGATATGAACGTTGAGACTGCCGCTGAGTTATCAGCTGTGAACGACATTCTGGCGTCTATCGGTGAACCTCCGGTa                                                                                                                                                                                                                                <  1:179773/300‑1 (MQ=255)`  
                                                                                `cAACGGTCGCTGCTAGTCCCGAAGAGGCGAGTGTTACTTCAACAGAAGAAACCTTAACGCCAGCACAGGAGGCCGCACGCACCCGCGCTGCTAACAAAGCCCGAAAGGAAGCTGAGTTGGCTGCTGCCACCGCTGAGCAATCACTAGCATAACCCCTTGGGGCCTCTAAACGGGTCTTGAGTGGTTTTTTGCTGAAAGGAGGAACTATATGCGCTCATGCGATATGAACGTTGAGCCTGCCGCTGAGTTATCAGCTGTGAACGACATTCTGGCGTCTATCGTTGAACCTCCGGTATCAAcc                                                                                                                                                                                                                          >  1:209147/1‑300 (MQ=255)`  
                                                                                     `gTCGCTGCTAGTCCCGAAGAGGCGAGTGTTACTTCAACAGAAGAAACCTTAACGCCAGCACAGGAGGCCGCACGCACCCGCGCTGCTAACAAAGCCCGAAAGGAAGCTGAGTTGGCTGCTGCCACCGCTGAGCAATCACTAGCATAACCCCTTGGGGCCTCTAAACGGGTCTTGAGGGGTTTTTTGCTGAAAGGAGGAACTATATTCGCTCATGCGATATGAACGTTGAGACTGCCGCTGAGTTATCAGCTGTGAACGACATTCTGGCGTCTATCGGTGAAccctgtcccttatacacatc                                                                                                                                                                                                                     >  1:126555/1‑283 (MQ=255)`  
                                                                                     `gTCGCAGCTAGACCCGAGAAGGCGAGTGTTACTTCAACAGAAGAAACCTTAACGCCAGCACAGGAGGCCGCACGCACCAGCGCTGCTAACAAAGCCCGAAAGGAAGCTGAGTTGTCTGCTGCCACCGCTGAGCAATCACTAGCATAACCCCTTGGGGCCTCTAAACGGGTCTTGAGGGGTTTTTTGCTGAAAGGAGGAACTATATGCGCTCATGCGATATGAACGTTGAGACTGCCGCTGAGTTATCAGCTGTGAACGACATTCTGGCGTCGATCGGTGAACCTCCGGTATCAACGCTGGa                                                                                                                                                                                                                     <  1:55619/301‑1 (MQ=255)`  
                                                                                      `gcGCTGCTAGTCCCGAAGAGGCGAGTGTTACTTCAACAGAAGAAACCTTAACGCCAGCACAGGAGGCCGCACGCACCCGCGCTGCTAACAAAGCCCGAAAGGAAGCTGAGTTGGCTGCTGCCACCGCTGAGCAATCACTAGCATAACCCCTTGGGGCCTCTAAACGGGTCTTGAGGGGTTTTTTGCTGAAAGGAGGAACTATATGCGCTCATGCGATATGAACGTTGAGACTGCCGCTGAGTTATCAGCTGTGAACGACATTCTGGCGTCTATCGGTGAACCTCCGGTATCAACGCTGGaa                                                                                                                                                                                                                    <  1:121289/300‑1 (MQ=255)`  
                                                                                      `cagcggctAGTCCCGAAGAGGCGAGTGTTACTTCAACAGAAGAACCCTTAACGCCAGCACAGGAGGCCGCACGCACCCGCGCTGCTAACAAAGCCCGAAAGGAATCTGAGTTGGCTGCTGCCACCGCTGAGCAATCACTAGCATAACCCCTTGGGGCCTCTAAACGGGTCTTGAGGGGTTTTTTGCTGAAAGTAGGAACTATATGCGCTCATGCGATATGAACGTTGAGACTGCCGCTGAGTTATCAGCTGTGAACGACATTCTGGCGTCTATCGGTGAACCTCCGGTATCAACGCTGGaa                                                                                                                                                                                                                    <  1:291417/296‑1 (MQ=255)`  
                                                                                         `ctgctAGTCCCGAAGAGGCGAGTGTTACTTCAACAGAAGAAACCTTAACGCCAGCACAGGAGGCCGCACGCACCCGCGCTGCTAACAAAGCCCGAAAGGAAGCTGAGTTGGCTGCTGCCACAGCTGAGCAATCACTAGCATAACCCCTTGGGGCCTCTAAACGGGTCTTGAGGGGTTTTTTGCTGAAAGGAGGAACTATATGCGCTCATGCGATATGAACGTTGAGACTGCAGCTGAGTTATCAGCTGTGAACGACATTCTGGCGTCTATCGGTGAACCTACGGTATCAACGCTGGAAGGt                                                                                                                                                                                                                 >  1:48312/1‑301 (MQ=255)`  
                                                                                           `gctAGTCCCGAAGAGGCGAGTGTTACTTCAACAGAAGAAACCTTAACGCCAGCACAGGAGGCCGCACGCACCCGCGCTGCTAACAAAGCCCGAAAGGAAGCTGAGTTGGCTGCTGCCACCGCTGAGCAATCACTAGCATAACCCCTTGGGGCCTCTAAACGGGTCTTGAGGGGTTTTTTGCTGAAAGGAGGAACTATATGCGCTCATGCGATATGAACGTTGAGACTGCCGCTGAGTTATCAGCTGTGAACGACATTCTGGCGTCTATCGGTGAACCTCCGGTATCAACGCTGGAAGGTGa                                                                                                                                                                                                               >  1:339998/1‑301 (MQ=255)`  
                                                                                                  `ccGAAGAGGCGAGTGTTACTTCAACAGAAGAAACCTTAACGCCAGCACAGGAGGCCGCACGCACCCGCGCTGCTAACAAAGCCCGAAAGGAAGCTGAGTTGGCTGCTGCCACCGCTGAGCAATCACTAGCATAACCCCTTGGGGCCTCTAAACGGGACTTGAGGGGTTTTTTGCTGAAAGGAGGAACTATATGCGCTCATGCGATATGAACGTCGAGACTGCCGCTGAGTTATCAGCTGTGAACGACATTCTGGCGTCTATCGGTGAACCTCCGGTATCAACGCTGGaactgtctcttata                                                                                                                                                                                                        >  1:6504/1‑289 (MQ=255)`  
                                                                                                    `gAAGAGGCGAGTGTTACTTCAACAGAAGAAACCTTAACGCCAGCACAGGAGGCCGCACGCACCCGCGCTGCTAACAAAGCCCGAAAGGAAGCTGAGTTGGCTGCTGCCACCGCTGAGCAATCACTAGCATAACCCCTTGGGGCCTCTAAACGGGTCTTGAGGGGTTTTTTGCTGAAAGGAGGAACTATATGCGCTCATGCGATATGAACGTTGAGACTGCCGCTGAGTTATCAGCTGTGAACGACATTCTGGCGTCTATCGGTGAACCTCCGGTATCAACGCTGGAAGGTGACGCTAACGc                                                                                                                                                                                                      >  1:94150/1‑301 (MQ=255)`  
                                                                                                      `agagGCGAGTGTTACTTCAACAGAAGAAACCTTAACGCCAGCACAGGAGGCCGCACGCACCCGCGCTGCTAACAAAGCCCGAAAGGAAGCTGAGTTGGCTGCTGCCACCGCTGAGCAATCACTAGCATAACCCCTTGGGGCCTCTAAACGGGTCTTGAGTGGTTTTTTGCTGAAAGGAGGAACTATATGCGCTCATGCGATATGAACGTTGAGACTGCCGCTGAGTTATCAGCTGTGAACGACATTCTGGCGTCTATCGGTGAACCTCCGGTATCAACGCTGGAAGGTGACGCTAACGCAg                                                                                                                                                                                                    <  1:23682/301‑1 (MQ=255)`  
                                                                                                              `gtgtTACTTCAACAGAAGAAACCTTAACGCCAGCACAGGAGGCCGCACGCACCCGCGCTGCTAACAAAGCCCGAAAGGAAGCTGAGTTGGCTGCTGCCACCGCTGAGCAATCACTAGCATAACCCCTTGGGGCCTCTAAACGGGTCTTGAGGGGTTTTTTGCTGAAAGGAGGAACTATATGCGCTCATGCGATATGAACGTTGAGACTGCCGCTGAGTTATCAGCTGTGAACGACATTCTGGCGTCTATCGGTGAACCTCCGGTATCAACGCTGGAAGGTGACGCTAACGCAGATGCAGCg                                                                                                                                                                                            >  1:1005/1‑301 (MQ=255)`  
                                                                                                                                `aaaCCTTAACGCCAGCACAGGAGGCCGCACGCACCCGCGCTGCTAACAAAGCCCGAAAGGAAGCTGAGTTTGCGGCTGCCACTGCTGAGCAATCACTAGCATAACCCCTTGGGGCCTCTAAACGGGTCTTGAGGGGTTTTTTGCTGAAAGGAGGAACTATATGTGCTCATGCGATATGAACGTTGAGACTGCCGCTGAGTTATCAGCTGTGAACGACATTCTGGCGTCTATCGGTGAACCTCCGGTATCAACGCTGGAAGGTGACGCTAACGCAGATGCAGCGAACGCTCGGCGTATtct‑c                                                                                                                                                                         <  1:138373/301‑1 (MQ=255)`  
                                                                                                                                `aaaCCTTAACGCCAGCACAGGAGGCCGCACGCACCCGCGCTGCTAACAAAGCCCGAAAGGAAGCTGAGTGGGCTGCTGCTACCGCTGAGCAATCACTAGCATAACCCCTTGGGGCCTCTAAACGGGTATTGAGGGGTTTTTTGCTGAAAGGAGGAACTATATGCGCTCATGCGATATGAACGTTGAGACTGCCGCTGAGTTATCAGCTGTGAACGACATTCTGGCGTCTATCGGTGAACCTCCGGTATCAACGCTGGAAGGTGACGCTAACGCAGATGCAGCGAACGCTCGGCGTATtct‑c                                                                                                                                                                         <  1:211468/301‑1 (MQ=255)`  
                                                                                                                                   `ccTTAACGCCAGCACAGGAGGCCGCACGCACCCGCGCTGCTAACAAAGCCCGAAAGGAAGCTGAGTTGGCTGCTGCCACCGCTGAGCAATCGCTAGCATAACCCCTTGGGGCCTCTAAACGGGTCTTGAGGGGTTTTTTGCTGAAAGTAGGAACTATATGCGCTCATGCGATATGAACGTTGAGACTGCCGCTGAGTTATCAGCTGTGAACGACATTCTGGCGTCTATCGGTGAACCTCCGGTATCAACGCTGGAAGGTGACGCTAACGCAGATGCAGCGAACGCTCGGCGTATTCT‑caac                                                                                                                                                                      <  1:42273/301‑1 (MQ=255)`  
                                                                                                                                   `ccTTAACGCCAGCACAGGAGGCCGCACGCACCCGCGCTGCTAACAAAGCCCGAAAGGAAGCTGAGTTGGCTGCTGCCACCGCTGAGCAATCACTAGCATAACCCCTTGGGGCCTCTAAACGGGTCTTGAGGGGTTTTTTGCTGAAAGGAGGAACTATATGCGCTCATGCGATATGAACGTTGAGACTGCCGCTGAGTTATCAGCTGTGAACGACATTCTGGCGTCTATCGGTGAACCTCAGGTATCAACGCTGGAAGGTGACGCTAACGCAGATGCAGCGAACGCTCGGCGTATTCT‑caac                                                                                                                                                                      <  1:79147/301‑1 (MQ=255)`  
                                                                                                                                         `cGCCAGCACAGGAGGCCGCACGCACCCGCGCTGCTAACAAAGCCCGAAAGGAAGCTGAGTTGGCTGCTGCCACCGCTGAGCAATCACTAGCATAACCCCTTGGGGCCTCTAAACGGGTCTTGAGTGGATTTTTGCTGCAAGGAGGAACTATATGCGCTCATGCGAGATGAACGTTGAGACTGCCGCTGAGTTATCAGCTGTGAACGACATTCTGGCGTCTATCGGTGAACCGCAGGTATCAACGCTGGAAGGTGACGCTAACGCAGATGCAGCGAACGCTCGGCGTATTCT‑caacctgtct                                                                                                                                                                >  1:140481/1‑295 (MQ=255)`  
                                                                                                                                              `gCACAGGAGGCCGCACGCACCCGCGCTGCTAACAAAGCCCGAAAGGAAGCTGAGTTGGCTGCTGCCACCGCTGAGCAATCACTAGCATAACCCCTTGGGGCCTCTAAACGGGTCTTGAGGGGTTTTTTGCTGAAAGGAGGAACTATATGCGCTCATGCGATATGAACGTTGAGACTGCCGCTGAGTTATCAGCTGTGAACGACATTCTGGCGTCTATCGGTGAACCTCCGGTATCAACGCTGGAAGGTGACGCTAACGCAGATGCAGCGAACGCTcctgtctcttatacacatctccgagc                                                                                                                                                            >  1:113415/1‑276 (MQ=255)`  
                                                                                                                                                       `gCCGCACGCACCCGCGCTGCTAACAAAGCCCGAAAGGAAGCTGAGTTGGCTGCTGCCACCGCTGAGCAATCACTAGCATAACCCCTTGGGGCCTCTAAACGGGTCTTGAGGGGGTTTTTGCTGAAAGGAGGAACTATATGCGCTCATGCGATATGAACGTTGAGACTGCCGCTGAGTTATCAGCTGTTAACGACATTCTGGCGTCTATCGGTGAACCTCCGGTATCAACGCTGGAAGGTGACGCTAACGCAGATGCAGCGAACGCTCGGCGTATTCC‑CAACAAGATTAAGCGACAGATTCa                                                                                                                                                  >  1:322629/1‑301 (MQ=255)`  
                                                                                                                                                          `gtgtataagagacaggctAACAAAGCCCGAAAGGAAGCTGAGTTGGCTGCTGCCACCGCTGAGCAATCACTAGCATAACCCCTTGGGGCCTCTAAACGGGTCTTGAGGGGTTTTTTGCTGAAAGGAGGAACTATATGCGCTCATGCGATATGAACGTTGAGACTGCCGCTGAGTTATCAGCTGTGAACGACATTCTGGCGTCTATCGGTGAACCTCCGGTATCAACGCTGGAAGGTGACGCTAACGCAGATGCAGCGAACGCTCGGCGTATTCT‑CAACAAGATTAACCGACAGATTCAAtc                                                                                                                                               <  1:103569/286‑1 (MQ=255)`  
                                                                                                                                                          `gcacgcacCCGCGCTGCTAACAAAGCCCGAAAGGAAGCTGAGTTGGCTGCTGCCACCGCTGAGCAATCACTAGCATAACCCCTTGGGGCCTCTAAACGGGTCTTGAGGGGTTTTTTGCTGAAAGGAGGAACTATATGCGCTCATGCGATATGAACGTTGAGACTGCCGCTGAGTTATCAGCTGTGAACGACATTCTGGCGTCTATCGGTGAACCTCCGGTATCAACGCTGGAAGGTGACGCTAACGCAGATGCAGCGAACGCTCGGCGTATTCT‑CAACAAGATTAACCGACAGATTCAAtc                                                                                                                                               <  1:314798/301‑1 (MQ=255)`  
                                                                                                                                                               `cacCCGCGCTGCTAACAAAGCCCGAAAGGAAGCTGAGTTGGCTGCTGCCACCGCTGAGCAATCACTAGCATAACCCCTTGGGGCCTCTAAACGGGTCTTGAGGGGTTTTTTGCTGAAAGGAGGAACTATATGCGCTCATGCGATATGAACGTTGAGACTGCCGCTGAGTTATCAGCTGTGAACGACATTCTGGCGTCTATCGGTGAACCTCCGGTATCAACGCTGGAAGGTGACGCTAACGCAGATGCAGCGAACGCTCGGCGTATTCT‑CAACAAGATTAACCGACAGATTCAAtctctgt                                                                                                                                          >  1:216281/1‑298 (MQ=255)`  
                                                                                                                                                               `cacCCGCGCTGCTAACAAAGCCCGAAAGGAAGCTGAGTTGGCTGCTGCCACCGCTGAGCAATCACTAGCATAACCCCTTGGGGCCTCTAAACGGGTCTTGAGGGGTTTTTTGCTGAAAGGAGGAACTATATGCGCTCATGCGATATGAACGTTGAGACTGCCGCTGAGTTATCAGCTGTGAACGACATTCTGGCGTCTATCGGTGAACCTCCGGTATCAACGCTGGAAGGTGACGCTAACGCAGATGCAGCGAACGCTCGGCGTATTCT‑CAACAAGATTAACCGACAGATTCAATCTCGtg                                                                                                                                          >  1:33726/1‑301 (MQ=255)`  
                                                                                                                                                                  `ccGCGCGGCTAAAAAAGCCCGAAAGGAAGCTGAGTTGGCTGCAGCCACCGATGAGCAATCACTAGCATAACCCCTTGGGGCCTCTAAACGGGTCTTGAGGGGTTTTTTGCTGAAAGGAGGAACTATATGCGCTCATGCGATATGAACGTTGAGACTGCCGCTGAGTTATCAGCTGTGAACGACATTCTGGCGTCTATCGGTGAACCTCCGGTATCAACGCTGGAAGGTGACGCTAACGCAGATGCAGCGAACGCTCGGCGTATTCT‑CAACAAGATTAACCGACAGATTCAATCTCGtggat                                                                                                                                       <  1:15241/301‑1 (MQ=255)`  
                                                                                                                                                                   `cgcgCTGCTAACAAAGCCCGAAAGGAAGCTGAGTTGGCTGCTGCCACCGCTGAGCAATCACTAGCATAACCCATTGGGGCCTCTAAACGGGTCTTGAGGGGTTTTTTGCTGAAAGGAGGAACTATATGCGCTCATGCGATATGAACGTTAAGACTGCCGCTGAGTTATCAGCTGTGAACGACATTCTGGCGTCTATCGGTGAACGTCCGGTATCAACGCTGGGAGGTGAAGCTAACGCAGATGCAGCGAACGCTCGGCATATTCT‑CAACATGAGTAACCGACAGATTCAATCTCGtgtatg                                                                                                                                      >  1:246969/1‑301 (MQ=255)`  
                                                                                                                                                                               `aaaGCCCGAAAGGAAGCTGAGTTGGCTGCTGCCACCGCTGAGCAATCACTAGCATAACCCCTTGGGGCCTCTAAACGGGTCTTGAGGGGTTTTTTGCTGAAAGGAGGAACTATATGCGCTCATGCGATATGAACGTTGAGACTGCCGCTGAGTTATCAGCTGTGAACGACATGCTGGCGTCTATCGGTGAACCTCCGGTATCAACGCTGGAAGGTGACGCTAACGCAGATGCAGCGAACGCTCGGCGTATTCT‑CAACAAGATTAACCGACAGATTCAATCTCGTGGATGGACGTTCAACAt                                                                                                                          <  1:284338/301‑1 (MQ=255)`  
                                                                                                                                                                                  `gCCCGAAAGGAAGCTGAGTTGGCTGCTGCCACCGCTGAGCAATCACTAGCATAACCCCTTGGGGCCTCTAAACGGGTCTTAAGGGGTTTTTTGCTGAAAGGAGGAACTATATGCGCTCATGCGATATGAACGTTGAGACTGCCGCTGAGTTATCAGCTGTGAACGACATTCTGGCGTCTATCGGTGAACCTCCGGTATCAACGCTGGAAGGTGACGCTAACGCAGATGCAGCGAACGCTCGGCGTATTCT‑CAACAAGATTAACCGACAGATTCAATCTCGTGGATGGACGTTCAACATTGa                                                                                                                       >  1:133360/1‑301 (MQ=255)`  
                                                                                                                                                                                         `aGGAAGCTGAGTTGGCTGCTGCCACCGCTGAGCAATCACTAGCATAACCCCTTGGGGCCTCTAAACGGGTCTTGAGTGGTTTTTTGCTGAAAGGAGGAACTATATGCGCTCATGTGATATGAACGTTGAGACTGCCGCTGAGTTATCAGCTGTGAACGACATTCTGGCGTCTATCGGTGAACCTCCGGTATCAACGCTGGAAGGTGACGCTAACGCAGATGCAGCGAACGCTCGGCGTATTCT‑CAACAAGATTAACCGACAGATTCAATCTCGTGGATGGACGTTCAACATTGAGGAAGGc                                                                                                                <  1:220140/301‑1 (MQ=255)`  
                                                                                                                                                                                          `ggAAGCTGAGTTGGCTGCTGCCACCGCTGAGCAATCACTAGCATAACCCCTTGGGGCCTCTAAACGGGTCTTGAGGGGTTTTTTGCTGAAAGGAGGAACTATATGCGCTCATGCGATATGAACGTTGAGACTGCCGCTGAGTTATAAGCTGTGCACGACATTCTGGCGTCTATAGGTGAACCTCCGGTATCAACGCTGGAAGGTGACGCTAACGCAGATGCAGCGAACGATCGGCGTATTCT‑ACACAAGATTAACCGACAGATTCAATCTCGTGGATGGACGTTCAACATTGAGGACGGCa                                                                                                               >  1:73482/1‑301 (MQ=255)`  
                                                                                                                                                                                          `gagacaggAGTTGGCTGCTGCCACCGCTGAGCAATCACTAGCATAACCCCTTGGGGCCTCTAAACGGGTCTTGAGGGGTTTTTTGCTGAAAGGAGGAACTATATGCGCTCATGCGATATGAACGTTGAGACTGCCGCTGAGTTATCAGCTGTGAACGACATTCTGGCGTCTATCTGTGAACCTCCGGTATCAACGCTGGAAGGTGACGCTAACGCAGATGCAGCGAACGCTCGGCGTATTCT‑CAACAAGATTAACCGACAGATTCAATCTCGTGGATGGACGTTCAACATTGAGGAAGGCa                                                                                                               <  1:97217/294‑1 (MQ=255)`  
                                                                                                                                                                                               `gggAGTTGGCTGCTGCCACCGCTGAGCAATCACGAGCATAACCCCTTGGGGCCTCTAAACGGGTCTTGAGGGGTTTTTTGCTGAAAGGAGGAACTATATGCGCTCATGCGATATGAACGTTGAGACTGCCGCTGAGTTATCAGCTGTGAACGACATTCTGGCGTCTATCGGTGAACCTCCGGTATCAACGCTGGAAGGTGACGCTAACGCAGATGCAGCGAACGCTCGGCGTATTCT‑CAACAAGATTAACCGACAGATTCAATCTCGTGGATGGACGTTCAACATTGAGGAAGGCATAACg                                                                                                          <  1:307773/299‑1 (MQ=255)`  
                                                                                                                                                                                                 `gAGTTGGCTGCTGCCACCGCTGAGCAATCACTAGCATAACCCCTTGGGGCCTCTAAACGGGTCTTGAGGGGTTTTTTGCTGCAAGGAGGAACTATATGCGCTCATGCGATATGAACGTTGAGACTGCCGCTGAGTTATCAGCTGTGAACGACATTCTGGCGTCTATCGGTGAACCTCCGGTATCAACGCTGGAAGGTGAAGCTAACGCAGAGGCAGTGAACGCTCGGCGTATTCT‑CAACAAGATTAACCGACAGATTAAATAGCGGGGAGGGACGTTCAACATTGCGCAAGGCATAACgat                                                                                                        >  1:27001/1‑299 (MQ=255)`  
                                                                                                                                                                                                        `ctgctgCCACCGCTGAGCAATCACTAGCATAACCCCTTGGGGCCTCTAAACGGGTCTTGAGGGGTTTTTTGCTGAAAGGAGGAACTATATGCGCTCATGCGATATGAACGTTGAGACTGCCGCTGAGTTATCAGCTGTGAACGACATTCTGGCGTCTATCGGTGAACCTCCGGTATCAACGCTGGAAGGTGACGCTAACGCAGATGCAGCGAACGCTCGGCGTATTCT‑CAACAAGATTAACCGACAGATTCAATCTCGTGGATGGACGTTCAACATTGAGGAAGGCATAACGCTACTACCt                                                                                                 <  1:206123/301‑1 (MQ=255)`  
                                                                                                                                                                                                          `gctgcCACCGCTGAGCAATCACTAGCATAACCCCTTGGGGCCTCTAAACGGGTCTTTAGGGGTTTTTTGCTGAAAGGAGGAACTATATGCGCTCATGCGATATGAACGTTGAGACTGCCGCTGAGTTATCAGCTGTGAACGACATTCTGGCGTCTATCGGTGAACCTCCGGTATCAACGCTGGAAGGTGACGCTAACGCAGATGCAGCGAACGCTCGGCGTATTCT‑CAACAAGATTAACCGACAGATTCAATCTCGTGGATGGACGTTCAACATTGAGGAAGGCATAACGCTACTACCTGa                                                                                               >  1:160248/1‑301 (MQ=255)`  
                                                                                                                                                                                                                   `gacaggCAATCACTAGCATAACCCCTTGGGGCCTCTAAACGGGTCTTGAGGGGTTTTTTGCTGAAAGGAGGAACTATATGCGCTCATGCGATATGAACGTTGAGACTGCCGCTGAGTTATCAGCTGTGAACGACATTCTGGCGTCTATCGGTGAACCTCCGGTATCAACGCTGGAAGGTGACGCTAACGCAGATGCAGCGAACGCTCGGCGTATTCT‑CAACAAGATTAACCGACAGATTCAATCTCGTGGATGGACGTTCAACATTGAGGAAGGCATAACGCTACTACCTGATGTTTACTc                                                                                      <  1:67612/296‑1 (MQ=255)`  
                                                                                                                                                                                                                   `gCTGAGCAATCACTAGCATAACCCCTTGGGGCCTCTAAACGGGTCTTGAGGGGTTTTTTGCTGAAAGGAGGAACTATATGCGCTCATGCGATATGAACGTTGAGACTGCCGCTGAGTTATCAGCTGTGAACGACATTCTGGCGTCTATCGGTGAACCTCCGGTATCAACGCTGGAAGGTGACGCTAACGCAGATGCAGCGAACGCTCGGCGTATTCT‑CAACAAGATTAACCGACAGATTCAATCTCGTGGATGGACGTTCAACATTGAGGAAGGCATAACGCTACTACCTGATGTTTACTc                                                                                      <  1:241185/301‑1 (MQ=255)`  
                                                                                                                                                                                                                    `cttatcagtcataatcATAACCTCTTGGGGCCCCTAAACGGTTCTTTAGGGGTTTTTGTCTTAACGGAGGACCTAGATGCGCTCATGCGTTATGAACGTTGAGACTTCCTCGGAGTTATCCGCTGTGCACGACATTCTGGCGTCTGTCGTTGAACCTCCGGTATCAACGCTGTAAGGTGACGCTAACGCAGATGCAGCGAACGCTCGGCGTATTAT‑CAACAAGGTTAACCGACATATTCAATCTCGTTTATGGACGTTCAACATTGAGGAAGGCATAACGCTACTACCTGATGTTTACTcc                                                                                     <  1:145695/286‑1 (MQ=255)`  
                                                                                                                                                                                                                    `cTGAGCAATCACTAGCATAACCCCTTGGGGCCTCTAAACGGGTCTTGAGGGGTTTTTTGCTGAAAGGAGGAACTATATGCGCTCATGCGATATGAACGTTGAGACTGCCGCTGAGTTATCAGCTGTGAACGACATTCTGGCGTCTATCGGTGAACCTCCGGTATCAACGCTGGAAGGTGACGCTAACGCAGATGCAGCGAACGCTCGGCGTATTCT‑CAACAAGATTAACCGACAGATTCAATCTCGTGGATGGACGTTCAACATTGAGGAAGGCATAACGCTACTACCTGATGTTTACTcc                                                                                     <  1:305110/301‑1 (MQ=255)`  
                                                                                                                                                                                                                        `taagagacaggCATAACCCCGTGGGGCCTCTAAACGGGTCTTGAGGGGTTTTTTGCTGAAAGGAGGAACTATATGCGCTCATGCGATATGAACGTTGAGACTGCCGCTGAGTTATCAGCTGTGAACGACATTCTGGCGTCTATCGGTGAACCTCCGGTATCAACGCTGGAAGGTGACGCTAACGCAGATGCAGCGAACGCTCGGCGTATTCT‑CAACAAGATTAACCGACAGATTCAATCTCGTGGATGGACGTTCAACATTGAGGAAGGCATAACGCTACTACCTGATGTTTACTCCAAcc                                                                                 <  1:221864/291‑1 (MQ=255)`  
                                                                                                                                                                                                                        `gCAATCACTAGCATAACCCCTTGGGGCCTCTAAACGGGTCTTGAGGGGTTTTTTGCTGAAAGGAGGATCTATATGCGCTAATGCGATATGAACGTTGAGACTGCCGCTGAGTTATCAGCTGTGAACGACATTCTGGCGTCTATCGGTGAACCTCCGGTATCAACGCTGGAAGGTGACGCTAACGCAGATGCAGCGAACGCTCGGCGTATTCT‑CAACAAGATTAACCGACAGATTCAATCTCGTGGATGGACGTTCAACATTGAGGAAGGCATAACGCTACTACCTGATGTTTACTCCAAcc                                                                                 <  1:76281/301‑1 (MQ=255)`  
                                                                                                                                                                                                                         `cAATCACTAGCATAACCCCTTGGGGCCTCTAAACGGGTCTTGAGGGGTTTTTTGCTGAAAGGAGGAACTATATGCGCTCATGCGATATGAACGTTGAGACTGCCGCTGAGTTATCAGCTGTGAACGACATTCTGGCGTCTATCGGTGAACCTCCGGTATCAACGCTGGAAGGTGACGCTAACGCAGATGCAGCGAACGCTCGGCGTATTCT‑CAACAAGATTAACCGACAGATTCAATCTCGTGGATGGACGTTCAACATTGAGGAAGGCATAACGCTACTACCTGATGTTTACTCCAACCt                                                                                >  1:156966/1‑301 (MQ=255)`  
                                                                                                                                                                                                                             `caCTAGCATAACCCCTTGGGGCCTCTAAACGGGTCTTGAGGGGTTTTTTGCTGAAAGGAGTAACTATATGCGCTCATGCGATATGAACGTTGAGACTGCCGCTGAGTTATCAGCTGTGAACGACATTCTGGCGTCTATCGGTGAACCTCCGGTATCAACGCTGGAAGGTGACGCTAACGCAGATGCAGCGAACGCTCGGCGTATTCT‑CAACAAGATTAACCGACAGATTCAATCTCGTGGATGGACGTTCAACATTGAGGAAGGCATAACGCTACTACCTGATGTTTACTcctgtctcttt                                                                            >  1:299452/2‑292 (MQ=255)`  
                                                                                                                                                                                                                             `caCTAGCATAACCCCTTGGGGCCTCTAAACGGGTCTTGAGGGGTTTTTTGCTGAAAGGAGGAACTATATGCGCTCATGCGATATGAACGTTGAGACTGCCGCTGAGTTATCAGCTGTGAACGACATTCTGGCGTCTATCGGTGAACCTCCGGTATCAACGCTGGAAGGTGACGCTAACGCAGATGCAGCGAACGCTCGGCGTATTCT‑CAACAAGATTAACCGACAGATTCAATCTCGTTGATGGACGTTCAACATTTAGGAAGGCATAACGCTACTACCTGATGTTTACTCCAACCTGAtt                                                                            >  1:280763/2‑301 (MQ=255)`  
                                                                                                                                                                                                                             `caCTAGCATAACCCCTTGGGGCCTCTAAACGGGTCTTGAGGGGTTTTTTGCTGAAAGGAGGAACTATATGCGCTCATGCGATATGAACGTTGAGACTGCCGCTGAGTTATCAGCTGTGAACGACATTCTGGCGTCTATCGGTGAACCTCCGGTATCAACGCTGGAAGGTGACGCTAACGCAGATGCAGCGAACGCTCGGCGTATTCT‑CAACAAGATTAACCGACAGATTCAATCTCGTGGATGGACGTTCAACATTGAGGAAGGCATAACGCTACTACCTGATGTTTACTctgtctcttat                                                                            >  1:106816/2‑291 (MQ=255)`  
                                                                                                                                                                                                                             `caCTAGCATAACCCCTTGGGGCCTCTAAACGGGTATTGAGGGGTTTTTTGCTGAAAGGAGGAACTATATGCCCCCATGCGTTATGACCGTTGCGACTGCCGTTGAGTTACCAGCTGTGAACGACATTCTGGCGTCTTTCGGTGAACCCCCGGTACCAACGCTGGAAGGTGACGCTAACGCAGATGCAGCGACCGATCGGCGT‑TTCTCCAACAAGATTAACCGCCAGTTCCAACCTCGTGGATGGCCGTTCATCATTGAGGAAGGCATAACGCACCTACCTGTTGTTTACTCCAACCTGAtt                                                                            >  1:294307/2‑301 (MQ=255)`  
                                                                                                                                                                                                                               `cTAGCATAACCCCTTGGGGCCTCTAAACGGGTCTTGAGGGGTTTTTTGCTGAAAGGAGGAACTATATGCGCTCATGCGATATGAACGTTGAGACTGCCGCTGAGTTATCAGCTGTGAACGACATTCTGGCGTCTATCGGTGAACCTCCGGTATCAACGCTGGAAGGTGACGCTAACGCAGATGCAGCGAACGCTCGGCGTATTCT‑CAACAAGATTAACCGACAGATTCAATCTCGTGGATGGACGTTCAACATTGAGGAAGGCATAACGCTACTACCTGATGTTTACTCCAACCTGATTGt                                                                          >  1:288049/1‑301 (MQ=255)`  
                                                                                                                                                                                                                                  `gCATAACCCCTTGTGGCCTCTAAACGGGTCTTGAGGGGTTTTTTGCTGAAAGGAGGAACTATATGCGCTCATGCGATATGAACGTTGAGACTGCCGCTGAGTTATCAGCTGTGAACGACATTCTGGCGTCTATCGGTGAACCTCCGGTATCAACGCTGGAAGGTGACGCTAACGCAGATGCAGCGAACGCTCGGCGTATTCT‑CAACAAGATTAACCGACAGATTCAATCTCGTGGATGGACGTTCAACATTGAGGAAGGCATAACGCTACTACCTGATGTTTACTCCAACCTGATTgctgt                                                                       >  1:165378/1‑297 (MQ=255)`  
                                                                                                                                                                                                                                  `gCATAACCCCTTGGGGCCTCTAAACGGGTCTTGAGGGGTTTTTTGCTGAACGGAGGAACTATATGCGCTCATGCGATATGAACGTTTAGACTGCCGCTGAGTTATCAGCTGTGAACGACATTCTGGCGTCTATCGGTGAACATCCGGTCTCAACGCTGGCAGGTGACGCTAACGCAGATGCAGCGAACGATCGGCGTATTCT‑CAACAAGATTAACCGACCGCTTCAATTTCATGGATGGACGTTCAACATTGAGGAAGGCATAACGCTACTCCCTGATGTTTCCTTCAACCTGATTGttta                                                                       >  1:325427/1‑301 (MQ=255)`  
                                                                                                                                                                                                                                  `gCATAACCCCTTGGGGCCTCTAAACGGGTCTTGAGGGGTTTTTTGCTGAAAGGAGGAACTATATGCGCTCATGCGATATTAACGTTGAGACTGCCGCTGAGTTATCAGCTGTGAACGACATTCTGGCGTCTATCGGTGAGCCTCCGGTATCAACGCTGGCAGGTGACGCTAACTCAGATTCAGCGAACGCTCGGCGTCTTCT‑CAACTAGATTAACCGACAGATTCACTCCCGTGGCTTGACTTTCAACATTTAGGAAGGCATACCCCTCCTACCTTATGTTTCCTCCAACCTGATTGcata                                                                       >  1:188035/1‑301 (MQ=255)`  
                                                                                                                                                                                                                                       `aCCCCTTGGGGCCTCTAAACGGGTCTTGAGTGGTTTTTTGCTGAAAGGAGGAACTATATGCGCTCATGCGATATGAACGTTGAGACTGCCGCTGAGTTATCAGCTGTGAACGACATTCTGGCGTCTATCGGTGAACCTCCGGTATCAACGCTGGAAGGTGACGCTAACGCAGATGCAGCGAACGCTCGGCGTATTCT‑CAACAAGATTAACCGACAGATTCAATCTCGTGGATGGACGTTCAACATTGAGGAAGGCATAACGCTACTACCTGATGTTTACTCAAACCTGATTGTATAcctgt                                                                  >  1:266721/1‑297 (MQ=255)`  
                                                                                                                                                                                                                                       `aCCCCTTGGGGCCTCTAAACGGGTCTTGAGGGGTTTTTTGCTGAAAGGAGGATCTATATGCGCTCATGCGATATGAACGTTGAGACTGCCGCTGAGTTATCAGCTGTTAACGACATTCTGGCGTATATCGGTGAACCTCCGGTATCAACGCTGGAATGTGACGCTAACGCAAATGCCGCGAACGCTCGGCGTATTCT‑CAACAAGATTAACCGACAGATTCAATCTCGTGGATGGACGTTCAACCTTGAGGAATGCATAACGCTACTCCCTGATTTTTACTCCAACCTAAtttctgtctctt                                                                  >  1:329112/1‑291 (MQ=255)`  
                                                                                                                                                                                                                                           `cTTGGGGCCTCTAAACGGGTCTTGAGGGGTTTTTTGCTGAAAGGAGGAACTATATGCGCTCATGCGATATGAACGTTGAGACTGCCGCTGAGTTATCAGCTGTTAACGACATTCTGGCGTCTATCGGTGAACCTCCGGTATCAACGCTGGAAGGTGACGCTAACGCAGATGCAGCGAACGCTCGGCGTATTCT‑CAACAAGATTAACCGACAGATTCAATCTCGTGGATGGACGTTCAACATTGAGGAAGGCATAACGCTACTACCTGATGTTTACTCCAACCTGATTGTATACAGTgacgg                                                              <  1:297015/301‑2 (MQ=255)`  
                                                                                                                                                                                                                                                   `ctctAAACGGGTCTTGAGGGGTTTTTTGCTGAAAGGAGGAACTATATGCGCTCATGCGATATGAACGTTGAGACTGCCGCTGAGTTATCAGCTGTGAACGACATTCTGGCGTCTATCGGTGAACCTCCGGTATCAACGCTGGAAGGTGACGCTAACGCAGATGCAGCGAACGCTCGGCGTATTCT‑CAACAAGATTAACCGACAGATTCAATCTCGTGGATGGACGTTCAACATTTAGGAAGGCATAACGCTACTACCTGATGTTTACTCAAACCTGATTGTATACAGTGACGGCTATTTAt                                                      >  1:30254/1‑301 (MQ=255)`  
                                                                                                                                                                                                                                                     `ctAAACGGGTCTTGATGGGTTTTTTGCTGAAAGGAGGAACTATATGCGCTCATGCGATATGAACGTTGAGACTGCCGCTGAGTTATCAGCTGTGAACGACATTCTGGCGTCTATCGGTGAACCTCCGGTATCAACGCTGGAATGTGACGCTAACGCAGATGCAGCGAACGCTCTGCGTATTCT‑CAACAAGATTAACCGACAGATTCAATCTCGTGGATGGACGTTCAACATTGAGGAAGGCATAACGCTACTACCTGATGTTTACTCCAACCTGATTGTATAcctgtctcttatacacatt                                                    >  1:44498/1‑283 (MQ=255)`  
                                                                                                                                                                                                                                                     `ctAAACGGGTCTTGAGGGGTTTTTTGCTGACAGGAGGAACTATATGCGCTCATGCGATATGAACGTTGAGACTTCCGCTGAGTTATCAGCTGTGAACGACATTCTGGCGTCTATCGGTTAACCTCCGGTATCAACGCTGCAAGGTGACGCTAACGCAGATGCAGCGAACGCTCGCAGTATTCT‑CAACAATATTAACCGACAGATTCAATCTCGTGGATGGACGTTCAACATTGAGGAAGGCATAACGCTACTACCTGATGTTTACTCCAACCTGATTGTATACAGtctgtctcttatacac                                                    >  1:310652/1‑286 (MQ=255)`  
                                                                                                                                                                                                                                                     `ctAAACGGGTCTTGAGGGGTTTTTTGCTGAAAGGAGGAACTATATGCGCTCATGCGATATGAACGTTGAGACTGCCGCTGAGTTATCAGCTGTGAACGACATTCTGGCGTCTATCGGTGAACCTCCGGTTTCAACGCTGGAAGGTGACGCTAACGCAGATGCAGCGAACGCTCGGCGTATTCT‑CAACAAGATTAACCGACAGATTCAATCTCGTGGATGGACGTTCAACATTGAGGAAGGCATAACGCTACTACCTGATGTTTACTCCAACCTGATTGTATAcctgtctcttatacacatc                                                    >  1:278310/1‑283 (MQ=255)`  
                                                                                                                                                                                                                                                     `ctAAACGGGGCTTGAGGGGTTTTTTGCTGAAAGGAGGAACTATATGCGCTCATGCGATATGAACGTTGAGACTGCCGCTGAGTTATCAGCTGTGAACGACATTCTGACGTCTATCGGTGAACCTCCGGTATCAACGCTGGAAGGTGACGCTAACGCAGATGCAGCGAACGATCGGCGTATTGT‑CAACAAGATTAACCGACAGATTCAATCTCGTGGATGGACGTTCAACATTGAGGAAGGCATAACGCTACTACCTAATGTGTAATCCAACATGATTGTATACAGAGACGGCTATTTATcc                                                    >  1:33160/1‑301 (MQ=255)`  
                                                                                                                                                                                                                                                      `tAAACAGTTCTTGAGTGGTTTTTTGCTGAAAGGAGGAACTATATGCGCTCATGCGATATGAACGTTGAGACTGCCTCTGAGTTATCAGCTGTGAACGACATTCTGGCGTCTATCGGTGACCCTCCGGTATCAACGCTGGAAGGTGACGCTAACGCAGATGCAGCGAACGCTCGGCGTATTCT‑CAACAAGATTAACCGACAGATTCAATCTCGTGGATGGACGTTCAACATTGAGGAAGGCATAACGCTACTACCTGATGTTTACTCCAACCTGATTGTATACAGTGACGGCTATTTATccc                                                   <  1:183306/301‑1 (MQ=255)`  
                                                                                                                                                                                                                                                      `aaaaCGGGTCTTGAGGGGTTGTTTGCTGAAAGGAGGAACTATATGCGCTCATGCGATATGAACGTTGAGACTGCCGCTGAGTTATCAGCTGTGAACGACATTCTGGCGTCTATCGGTGAACCTCCGGTATCAACGCTGGAAGGTGACGCTAACGCAGATGCAGCGAACGCTCGGCGTATTCT‑CAACAAGATTAACCGACAGATTCAATCTCGTGGATGGACGTTCAACATTGAGGAAGGCATAACGCTACTACCTGATGTTTACTCCAACCTGATTGTATACAGTGACGGCTATTTAGccc                                                   <  1:130015/300‑1 (MQ=255)`  
                                                                                                                                                                                                                                                             `ggtgggctcggagatgtgtataagagacagaCTATATGCGCTCATGCGATATGAACGTTGAGACTGCCGCTGAGTTATCAGCTGTGAACGACATTCTGGCGTCTATCGGTGAACCTCCGGTATCAACGCTGGAAGGTGACGCTAACGCAGATGCAGCGAACGCTCGGCGTATTCT‑CAACAAGATTAACCGACAGATTCAATCTCGTGGATGGACGTTCAACATTGAGGAAGGCATAACGCTACTACCTGATGTTTACTCCAACCTGATTGTATACAGTGACGGCTATTTATCCCTAATGTc                                            <  1:62527/271‑1 (MQ=255)`  
                                                                                                                                                                                                                                                             `gTCTTGAGGGGTTTTTTGCTGAAAGGAGGAACTATATGCGCTCATTCGATATGAACGTTGAGACTGCCGCTGAGTTATCAGCTGTTAACGACATTCTGGCGTCTATCGGTGAACCTCCGGTATCAACGCTTGAAGGTGACGCTAACGCAGCTGCAGCGAACGCTCGGCGTATTCT‑CAACAAGATTAACCGACAGATTCAATCTCGTTGATGGACGTTCAACATTTAGGAAGGCATAACGCTACTACCTGATGTTTACTCCAACCTGATTGTATAcctgtctcttatacacatctccgcgcc                                            >  1:19552/1‑275 (MQ=255)`  
                                                                                                                                                                                                                                                                    `ggctcggagatgggtataagagacagatagGCGATCATGCGATATGAACGGTGAGACTGCCGCTGAGTTATCAGCTGTGAACGACATTCTGGCGTCTATCGGTGAACCTCCGGTATCAACGCTGGAAGGTGACGCTAACGCAGATGCAGCGAACGCTCGGCGTATTCT‑CAACAAGATTAACCGACAGATTCAATCTCGTGGATGGACGTTCAACATTGAGGAAGGCATAACGCTACTACCTGATGTTTACTCCAACCTGATTGTATACAGTGACGGCTATTTATCCCTAATGTCTACTTcc                                     <  1:136050/275‑1 (MQ=255)`  
                                                                                                                                                                                                                                                                       `gTTTTTTGCTGAAAGTAGGAACTATATGCGCTCATGCGATATGAACGTTGAGACTGCCGCTGAGTTATCAGCTGTGAACGACATTCTGGCGTCTATCGGTGAACCTCCGGTATCAAAGCTGGAAGGTGAGGCTAACGCAGATGCAGCGAACGCTCGGCGTATTCT‑AAACAAGATTAACCGACAGATTCAATCTCGTGGATGGACGTTCAACATTGTGGAAGGCATAACGCTACTACCTGATGTTTACTCCAACCTGATTGTATACAGTGACGGCTATTTATCCCTAATGTCTACTTcatgt                                  >  1:35048/1‑297 (MQ=255)`  
                                                                                                                                                                                                                                                                        `ttttttGCTGAAAGGAGGAACTAGATGCGCTCATGCGAGATGAACGTTGAGACTGCCGCTGAGTTATCAGCTGTGAACGACAGTCTGGCGTCTATCGGTGAACCTCCGGTATCAACGCTGGAAGGTGACGCTAACGCAGATGCAGCGAACGCTCGGCGTATTCT‑CAACAAGATTAACCGACAGATTCAATCTCGTGGATGGACGTTCAACATTGAGGAAGGCATAACGCTACTACCTGATGTTTACTCCAACCTGATTGTATACAGTAACGGCTATTTATCCCTAATGTCTACTTCCGGTc                                 <  1:263929/301‑1 (MQ=255)`  
                                                                                                                                                                                                                                                                                    `aggaggAACTATATGCGCTCATGCGATATGAACGTTGAGACTGCCGCTGAGTTATCAGCTGTGAACGACATTCTGGCGTCTATCGGTGAACCTCCGGTATCAACGCTGGAAGGTGACGCTAACGCAGATGCAGCGAACGCTCGGCGTATTCT‑CAACAAGATTAACCGACAGATTCAATCTCGTGGATGGACGTTCAACATTGAGGAAGGCATAACGCTACTACCTGATATTTACTCCAACCTGATTGTATACAGTGACGGCTATTTATCCCTAATGTCTACTTCCGGTCAATCCATCTACg                     >  1:5941/1‑301 (MQ=255)`  
                                                                                                                                                                                                                                                                                     `ggaggaACTATATGCGCTCATGCGATATGAACGTTGAGACTGCCGCTGAGTTATCAGCTGTGAACGACATTCTGGCGTCTATCGGTGAACCTCCGGTATCAACGCTGGAAGGTGACGCTAACGCAGATGCAGCGAACGCTCGGCGTATTCT‑CAACAAGATTAACCGACAGATTCAATCTCGTGGATGGACGTTCAACATTGAGGAAGGCATAACGCTACTACCTGATGTTTACTCCAACCTGATTGTATACAGTGACGGCTATTTATCCCTAATGTCTACTTCCGGTCAATCCATCTACGt                    <  1:123989/301‑1 (MQ=255)`  
                                                                                                                                                                                                                                                                                     `gagacagcTATATGCGCTCATGCGATATGAACGTTGAGACTGCAGCTGAGTTATCAGCTGTGAACGACATTCTGGCGTCTATCGGTGAACCTCCGGTATCAACGCTGGAAGGTGACGCTAACGCAGATGCAGCGAACGCTCGGCGTATTCT‑AAACAAGATTAACCGACAGATGCAATCTCGTGGATGGACGTTCAACATTGAGGAAGGCATAACGCTACTACCTGATGTTTACTCCAACCTGATTGTATACAGTGACGGCTATTTATCCCTAATGTCTACTTCCGGTCAATCCATCTACGt                    <  1:38985/294‑1 (MQ=255)`  
                                                                                                                                                                                                                                                                                     `acaggaACTATATGCGCTCATGCGATATGAACGGTGAGACTGCCGCTGAGTGATCAGCTGTGAACGACATTCTGGCGTCTATCGGTGAACCTCCTGTATCAACGCTGGAAGGTGACACTAACGCAGATGCAGCGAACGCTCGGCGTATTCT‑CAACAAGATTAACCGACAGATTCAATCTCGTGGATGGACGTTCAACATTGAGGAAGGCATAACGCTACTACCTGATGTTTACTCCAACCTGATTGTATACAGTGACGGCTATTTATCCCTAATGTCTACTTCCGGTCAATCCATCTACGt                    <  1:50839/299‑1 (MQ=255)`  
                                                                                                                                                                                                                                                                                      `gaggaACTATATGCGCTCATGCGATATGAACGTTGAGACTGCCGCTGAGTTATCAGCTGTGAACGACATTCTGGCGTCTATCGGTGAACCTCCGGTATCAACGCTGGAAGGTGACGCTAACGCAGATGCAGCGAACGCTCGGCGTATTCT‑CAACAAGATTAACCGACAGATTCAATCTCGTGGATGGACGTTCAACATTGAGGAAGGCATAACGCTACTACCTGATGTTTACTCCAACCTGATTGTATACAGTGACGGCTATTTATCCCTAATGTCTACTTCCTGTctcttatacacatct                   >  1:174977/1‑287 (MQ=255)`  
                                                                                                                                                                                                                                                                                        `ggaACTATATGCGCTCATGCGATATGAACGTTGAGACTGCCGCTGAGTTATCAGCTGTGAACGACATTCTGGCGTCTATCGGTGAACCTCCGGTATCAACGCTGGCAGGTGACGCTAACGCAGATGCAGCGAACGCTCGGCGTATTCT‑AAAGAAGATTAAACGACAGATTCACTACCGTGGATGGACGTTCAACATTGAGGAAGGCATAACGCTACTACCTGATGTTTACTCCAAAATGATTGTATACAGTGACGGCTATTTATCCATAATGTCTACTTCCGGTCATTCCATCTACGttca                 >  1:334115/1‑299 (MQ=255)`  
                                                                                                                                                                                                                                                                                        `ggaACTATATGCGCTCATGCGATATGAACGTTGAGACTGCCGCTGAGTTATCAGCTGTGAACGACATTCTGGCGTCTATCGGTGAACCTCCGGTATCAACGCTGGAAGGTGACGCTAACGCAGATGCAGCGAACGCTCGGCGTATTCT‑CAACAAGATTAACCGACAGATTCAATCTCGTGGATGGACGTTCAACATTGAGGAAGGCATAACGCTACTACCTGATGTTTACTCCAACCTGATTGTATACAGTGACGGCTATTTATCCCTAATGTCTACTTCCGGTCAATCCATCTACGTTaa                 >  1:214303/1‑301 (MQ=255)`  
                                                                                                                                                                                                                                                                                         `gaACTATATGCGCTCATGCGATATGAACGTTGAGACTGCCGCTGAGTTATCAGCTGTGAACGACATTCTGGCGTCTATCGGTGAACCTCCGGTATCAACGCTGGAAGGTGACGCTAACGCAGATGCAGCGAACGCTCGGCGTATTCT‑CAACAAGATTAACCGACAGATTCAATCTCGTGGATGGACGTTCAACATTGAGGAAGGCATAACGCTACTACCTTATGTTTACTCCAACCTGATTGTATACAGTGACGGCTATTTATCCCTAATGTCTACTTCCGGTCAATCCATCTACGTTAAc                >  1:203200/1‑301 (MQ=255)`  
                                                                                                                                                                                                                                                                                         `gaACTATATGCGCTCATGCGATATGAACGTTGAGACTGCCGCTGAGTTATCAGCTGTGAACGACATTCTGGCGTCTATCGGTGAACCTCCGGTATCAACGCTGGAAGGTGACGCTAACGCAGATGCAGCGAACGCTCGGCGTATTCT‑CAACAAGATTAACCGACAGATTCAATCTCGTGGATGGACGTTCAACATTGAGGAAGGCATAACGCTACTACCTGATGTTTACTCCAACCTGATTGTACACAGTGACGGCTATTTATCCCTAATGTCTACTTCCGGTCAATCCATCTACGTTAAc                >  1:227756/1‑301 (MQ=255)`  
                                                                                                                                                                                                                                                                                           `aCTATATGCGCTCATTCGATATGAACGTTGAGACTGCCGCTGAGTTATCAGCTGTGAACGACATTCTGGCGTCTATCGGTGAACCTCCGGTATCAACGCTGGAAGGTGACGCTAACGCAGATGCAGCGAACGCTCGGCGTATTCT‑CAACAAGATTAACCGACAGATTCAATCTCGTGGATGGACGTTCAACATTGAGGAAGGCATAACGCTACTACCTGATGTTTACTCCAACCTGATTGTATACAGTGACGGCTATTTATCCCTAATGTCTACTTCCGGTcctgtaccttatacacatct              >  1:29055/1‑282 (MQ=255)`  
                                                                                                                                                                                                                                                                                              `atatGCGCTCATGCGATATGAACGTTGAGACTGCCGCTGAGTTATCAGCTGTGAACGACATTCTGGCGTCTATCGGTGAACCTCCGGTATCAACGCTGGAAGGTGACGCTAACGCAGATGCAGCGAACGCTCGGCGTATTCT‑CAACAAGATTAACCGACAGATTCAATCTCGTGGATGTACGTTCAACATTGAGGAAGGCATAACGCTACTACCTGATGTTTACTCCAACCTGATTGTATACAGTGACGGCTATTTATCCCTAATGTCTACTTCCGGTCAATCCATCTACGTTAACCGAgg           >  1:149703/1‑301 (MQ=255)`  
                                                                                                                                                                                                                                                                                              `atatGCGCTCATGCGATATGAACGTTGAGACTGCCGCTGAGTTATCAGCTGTGAACGACATTCTGGCGTCTATCGGTGAACCTCCGGTATCAACGCTGGAAGGTGACGCTAACGCAGATGCAGCGAACGCTCGGCGTATTCT‑CAACAAGATTAACCGACAGATTCAATCTCGTGGATGGACGTTCAACATTGAGGAAGGCATAACGCTACTACCTGATGTTTACTCCAACCTGATTGTATACAGTGACGGCTATTTATCCCTAATGTCTACTTCCTGTctcttatacacatctccgagccc           >  1:230844/1‑279 (MQ=255)`  
                                                                                                                                                                                                                                                                                                    `gcTCATGCGATATGAACGTTGAGACTGCCGCTGAGTTATCAGCTGTGAACGACATTCTGGCGTCTATCGGTGAACCTCCGGTATCAACGCTGGAAGGTGACGCTAACGCAGATGCAGCGAACGCTCGGCGTATTCT‑CAACAAGATTAACCGACAGATTCAATCTCGTGGATGGACGTTCAACATTGAGGAAGGCATAACGCTACTACCTGATGTTTACTCCAACCTGATTGTATACAGTGACGGCTATTTATCCCTAATGTCTACTTCCGGTCAATCCATCTACGTTAACCGAGGTGGCTa     <  1:116667/301‑1 (MQ=255)`  
                                                                                                                                                                                                                                                                                                     `cTCATGCGATATGAACGTTGAGACTGCCGCTGAGTTATCAGCTGTGAACGACATTCTGGCGTCTATCGGTGAACCTCCGGTATCAACGCTGGAAGGTGACGCTAACGCAGATGCAGCGAACGCTCGGCGTATTCT‑CAACAAGATTAACCGACAGATTCAATCTCGTGGATGGACGTTCAACATTGAGGAAGGCATAACGCTACTACCTGATGTTTACTCCAACCTGATTGTATACAGTGACGGCTATTTATCCCTAATGTCTACTTCCGGTCAATCCATCTACGTTAACCGAGGTGGCTAt    >  1:22501/1‑301 (MQ=255)`  

                                                                                                                                                                                                                                                                                                          `|`                                                                                                                                                                                                                                                                                                            
`ACGCAATGGGCCACGGTGGTCTTCGCCCAGAAGCTGCTGGTGCAGTGGTTTTCAAAGTGGAGTAATGCTGGGGGTGGCCTCAACGGTCGCTGCTAGTCCCGAAGAGGCGAGTGTTACTTCAACAGAAGAAACCTTAACGCCAGCACAGGAGGCCGCACGCACCCGCGCTGCTAACAAAGCCCGAAAGGAAGCTGAGTTGGCTGCTGCCACCGCTGAGCAATAACTAGCATAACCCCTTGGGGCCTCTAAACGGGTCTTGAGGGGTTTTTTGCTGAAAGGAGGAACTATATGCGCTCATACGATATGAACGTTGAGACTGCCGCTGAGTTATCAGCTGTGAACGACATTCTGGCGTCTATCGGTGAACCTCCGGTATCAACGCTGGAAGGTGACGCTAACGCAGATGCAGCGAACGCTCGGCGTATTCT‑CAACAAGATTAACCGACAGATTCAATCTCGTGGATGGACGTTCAACATTGAGGAAGGCATAACGCTACTACCTGATGTTTACTCCAACCTGATTGTATACAGTGACGACTATTTATCCCTAATGTCTACTTCCGGTCAATCCATCTACGTTAACCGAGGTGGCTATGT  >  NC_001604.1/23940‑24535`

  

Alignment Legend

Aligned base mismatch/match (shaded by quality score): `ATCG/ATCG` `< 3 ≤` `ATCG/ATCG` `< 10 ≤` `ATCG/ATCG` `< 22 ≤` `ATCG/ATCG` `< 34 ≤` `ATCG/ATCG` `< 38 ≤` `ATCG/ATCG`

Unaligned base: `atcg`    Masked matching base: `atcg`    Alignment gap: `‑`    Deleted base: `‑`
